# Association of a Lay Health Worker Intervention With Symptom Burden, Survival, Health Care Use, and Total Costs Among Medicare Enrollees With Cancer

**DOI:** 10.1001/jamanetworkopen.2020.1023

**Published:** 2020-03-16

**Authors:** Manali I. Patel, David Ramirez, Richy Agajanian, Hilda Agajanian, Tumaini Coker

**Affiliations:** 1 Division of Oncology, Stanford University School of Medicine, Stanford, California; 2 Medical Services, Veterans Affairs Palo Alto Health Care System, Palo Alto, California; 3 Center for Primary Care and Outcomes Research/Health Research and Policy, Stanford University School of Medicine, Stanford, California; 4 CareMore Health, Cerritos, California; 5 The Oncology Institute of Hope and Innovation, Downy, California; 6 Seattle Children’s Research Institute, Seattle, Washington; 7 Department of Pediatrics, University of Washington School of Medicine, Seattle

## Abstract

**Importance:**

Undertreated patient symptoms require approaches that improve symptom burden.

**Objective:**

To determine the association of a lay health worker–led symptom screening and referral intervention with symptom burden, survival, health care use, and total costs among Medicare Advantage enrollees with a new diagnosis of solid or hematologic malignant neoplasms.

**Design, Setting, and Participants:**

This quality improvement study conducted at 9 community oncology practices from November 1, 2016, to October 31, 2018, compared newly diagnosed Medicare Advantage enrollees with solid or hematologic malignant neoplasms with patients diagnosed and treated 1 year prior. Analysis was conducted from August 1, 2019, to January 11, 2020.

**Interventions:**

Usual care augmented by a lay health worker trained to screen symptoms and refer patients to palliative care and behavioral medicine.

**Main Outcomes and Measures:**

The primary outcome was change in symptoms using the Edmonton Symptom Assessment Scale and the 9-item Patient Health Questionnaire at baseline and 6 and 12 months after enrollment. Secondary outcomes were between-group comparison of survival, 12-month health care use, and costs.

**Results:**

Among 425 patients in the intervention group and 407 patients in the control group, the mean (SD) age was 78.8 (8.3) years, 345 (41.5%) were female, and 407 (48.9%) were non-Hispanic white. Patients in the intervention group experienced a lower symptom burden as measured by the Edmonton Symptom Assessment Scale score over time compared with patients in the control group (mean [SD] difference, −1.9 [14.2]; 95% CI, −3.77 to −0.19; *P* = .01 for the intervention group and 2.32 [17.7]; 95% CI, 0.47 to 4.19; *P* = .02 for the control group). Similar findings were noted in 9-item Patient Health Questionnaire depression scores (mean [SD] difference, −0.63 [3.99]; 95% CI, −1.23 to −0.028; *P* = .04 for the intervention group and 1.67 [5.49]; 95% CI, 0.95 to 2.37; *P* = .01 for the control group). Patients in the intervention group compared with patients in the control group had fewer mean (SD) inpatient visits (0.54 [0.77]; 95% CI, 0.47-0.61 vs 0.72 [1.12]; 95% CI, 0.61-0.83; *P* = .04) and emergency department visits (0.43 [0.76]; 95% CI, 0.36-0.50 vs 0.57 [1.00]; 95% CI, 0.48-0.67; *P* = .002) per 1000 patients per year and lower total costs (median, $17 869 [interquartile range, $6865-$32 540] vs median, $18 473 [interquartile range, $6415-$37 910]; *P* = .02). A total of 180 patients in the intervention group and 189 patients in the control group died within 12 months. Among those who died, patients in the intervention group had greater hospice use (125 of 180 [69.4%] vs 79 of 189 [41.8%]; odds ratio, 3.16; 95% CI, 2.13-4.69; *P* < .001), fewer mean (SD) emergency department and hospital visits (emergency department: 0.10 [0.30]; 95% CI, 0.06-0.14 vs 0.30 [0.46]; 95% CI, 0.24-0.38; *P* = .001; hospital: 0.27 [0.44]; 95% CI, 0.21-0.34 vs 0.43 [0.82]; 95% CI, 0.32-0.55; *P* = .02), and lower costs (median, $3602 [interquartile range, $1076-$9436] vs median, $12 726 [interquartile range, $5259-$22 170]; *P* = .002), but there was no significant difference in inpatient deaths (18 of 180 [10.0%] vs 30 of 189 [15.9%]; *P* = .14).

**Conclusions and Relevance:**

This study suggests that a lay health worker–led intervention may be one way to improve burdensome and costly care.

## Key Points

**Question:**

Is a lay health worker–led screening and referral intervention associated with patient symptom burden, acute care use, and total costs of care?

**Findings:**

In this multisite quality improvement study of 425 Medicare Advantage enrollees with a diagnosis of cancer who were provided usual cancer care augmented by a lay health worker trained to proactively screen patient symptoms, discuss symptoms with a physician assistant, and refer patients with uncontrolled symptoms to palliative care and behavioral medicine compared with 407 control patients diagnosed and treated in the year prior, patients in the intervention group were associated with significant reductions in symptom burden over time, and patients in the control group were associated with worsening symptoms over time. Patients who received the intervention were associated with fewer inpatient and emergency department visits and lower median total costs, and there were no differences in survival.

**Meaning:**

The findings suggest that a lay health worker–led symptom screening and proactive referral intervention may improve value-based cancer care.

## Teaser

This quality improvement study examines the association of a lay health worker–led symptom screening and referral intervention with symptom burden, survival, health care use, and total costs among Medicare Advantage enrollees with a new diagnosis of solid or hematologic malignant neoplasms.

## Introduction

Despite efforts to improve symptoms among adults with cancer,^1,2,3,4^ suboptimal management of symptoms continues to affect many patients.^5,6,7,8^ Undertreated symptoms are associated with acute care use,^9^ inpatient deaths,^10^ psychological and physical distress,^11^ and financial burden to patients and the health care system.^12^ Barriers to appropriate management of symptoms include lack of infrastructure,^13,14^ clinician time,^15^ and reimbursement.^15,16^

A lay health worker (LHW)–led (ie, personnel who are not clinically trained) symptom screening intervention for adults with advanced cancer was previously developed.^17^ The goal was to proactively identify symptoms to prevent psychological and physical distress and decrease acute care use. The LHW was trained to assess symptoms and relay them to a supervising physician assistant (PA), who conducted any necessary interventions. Compared with patients who received a diagnosis of cancer the year prior, patients in the intervention group had better experiences of care, a better self-reported overall health status, a better self-reported mental and emotional health status, fewer emergency department (ED) visits and hospitalizations, lower health care costs, and no differences in hospice use compared with patients who were not in the intervention group.

In this study, we expanded the LHW-led intervention for all newly diagnosed patients regardless of cancer stage and implemented it across 9 community-based clinics. We also trained the LHW to refer patients with uncontrolled symptoms to palliative care and behavioral medicine, with the intended goal to improve symptom burden early and prevent acute care use. Although 1 prior study assessed the navigation of distress screening by LHWs,^18^ to our knowledge, there have been no studies that examine an LHW-led symptom screening and supportive services referral intervention across multiple community practices.

The primary objective of this study was to evaluate the association of this LHW-led symptom screening intervention with patient-reported symptom burden. The secondary objectives were to evaluate association of this LHW-led symptom screening intervention with survival, health care use, total health care costs, and end-of-life care among patients who died within 12 months of diagnosis.

## Methods

### Study Design and Population

We conducted a multisite quality improvement study using a pre-post design with CareMore Health, a Medicare Advantage health plan, and 9 of 10 oncology clinics within the Oncology Institute of Hope and Innovation in Los Angeles, California. We excluded 1 clinic because it was involved in the previously reported intervention.^17^ We enrolled all CareMore Medicare Advantage beneficiaries with newly diagnosed solid tumors or hematologic malignant neoplasms in these 9 clinics and excluded patients who did not receive medical oncology care. Patients were screened by a new patient coordinator, enrolled from November 1, 2016, to October 31, 2017, and followed up for 12 months after enrollment or until death, whichever was first. The comparison group (control group) was composed of CareMore Medicare Advantage beneficiaries with newly diagnosed solid tumors or hematologic malignant neoplasms who received medical oncology care at these same 9 clinics in the year prior to the intervention, from November 1, 2015, to October 31, 2016. The control group received usual cancer care without standardized symptom screening or management. We identified the control group through both CareMore claims data for all patients in these 9 clinics with 2016 *International Statistical Classification of Diseases and Related Health Problems, Tenth Revision, Clinical Modification* codes C00-D49, indicating receipt of cancer services during the dates of interest, and verified by the Oncology Institute of Hope and Innovation cancer registry, which contains data on patients’ names, payers, clinics, cancer stages, cancer diagnoses, symptom screening dates and scores, and diagnosis dates. The Stanford University Institutional Review Board reviewed and approved this study as quality improvement. Because the study was implemented as a process change for all patients in the clinic, the institutional review board waived formal patient consent. This study followed the Standards for Quality Improvement Reporting Excellence (SQUIRE) reporting guidelines.^19^

### Expanded LWH-Led Symptom Screening Intervention

The 12-month telephonic intervention consisted of 1 LHW who assessed symptoms using the validated Edmonton Symptom Assessment Scale (ESAS; score range, 0-10, where 0 indicates no symptoms and 10 indicates the worst possible symptoms)^20^ and screened for depression using the validated 9-item Patient Health Questionnaire (PHQ-9; score range, 0-27, where a score of 5 was a cutpoint for mild depression, a score of 10 was a cutpoint for moderate depression, a score of 15 was a cutpoint for moderately severe depression, and a score of 20 was a cutpoint for severe depression) (eAppendix 1 in the Supplement).^21^ All patients in both groups completed the ESAS and PHQ-9 at the first oncology visit after their cancer diagnosis and again at 6 and 12 months after diagnosis. The LHW contacted all eligible patients across the 9 clinic sites 1 week prior to their first oncology appointment. Patients were risk stratified into a high-risk category if they received a diagnosis of metastatic cancer, were undergoing active chemotherapy, or reported moderate or greater symptom burden (ESAS score ≥4) or moderate or greater depression risk (PHQ-9 score ≥10). All other patients were stratified into the low-risk category. The supervising PA risk stratified patients weekly and reassigned them based on changes in clinical characteristics. The LHW called patients in the high-risk category weekly and called those in the low-risk category monthly. The LHW documented all scores in the electronic health record and forwarded an encounter note to the PA and oncology clinician on the same day for review. To improve symptom relief for patients with uncontrolled symptoms identified by the LHW, the LHW proactively referred patients with a change in symptom scores of greater than 2 points in subsequent assessments to palliative care and to behavioral medicine if their PHQ-9 score reflected moderate depression. Telephone calls were conducted Monday through Friday between 8:30 am and 5:00 pm. The LHW also provided patients with basic education about palliative care services. The LHW had a Bachelor of Arts degree, had no prior clinical experience, worked 40 hours per week, and completed a 10-hour training led by the PA. The PA dedicated 25% full-time equivalent for this program, met in-person with the LHW once weekly to discuss patients, and reviewed all LHW documentation daily.

CareMore Health supported this intervention by providing financial support for the LHW, 25% full-time equivalent for the PA, and a $150 per member per month incentive to cover any additional costs, such as clinical time, incurred to the oncology clinicians for conducting the intervention. CareMore Health preauthorized all LHW referrals to palliative care and behavioral medicine.

### Outcomes

Prior to conducting the study, investigators, payer executives, and clinicians agreed to measure outcomes to determine the intervention’s success. The decision was not to measure intermediary variables (eg, referrals to palliative care) but to measure associations wtih patient-reported symptoms, healthcare use (ie, ED visits and hospitalizations), and total health care spending. A preplanned subset analysis evaluated the association with end-of-life care and costs (ie, ED visits, hospitalizations and total costs in the last month of life, hospice use, and acute care facility deaths) for the patients who died within 12 months of diagnosis. Process metrics included intervention deviations, percentage of patients contacted 1 week before their first oncology appointment, and mean number of LHW-patient contacts.

### Survival

We collected data on vital status and death dates from CareMore Health registrars. These data were validated by the Oncology Institute of Hope and Innovation cancer registry.

### Health Care Use and Health Care Costs

We collected data for the following cancer care services and total costs from CareMore Health claims data for all patients: palliative care use; behavioral medicine use; dates of all ED visits, hospital admissions, and hospital discharges; and the use of hospice services. We measured all health care use within the 12-month enrollment period (which also represented a 12-month postdiagnosis period) for the intervention group and the 12-month postdiagnosis period for the control group. We collected all claims data from CareMore Health to measure total costs, including inpatient and outpatient costs for the intervention and control groups. Using these same data sources for patients who died during the 12-month enrollment or postdiagnosis period, we measured ED visits, hospitalizations, and total costs in the last 30 days of life as well as acute care facility deaths.

### Demographic and Clinical Characteristics

We collected data on age, sex, self-reported race/ethnicity, cancer diagnosis, cancer stage, and clinic site from the electronic health record. We collected data on the Centers for Medicare & Medicaid Services Hierarchical Condition Category risk adjustment factor,^22^ which is based on a patient’s underlying health conditions, sex, age, Medicaid enrollment, and disabled status using CareMore Health claims data.

### Statistical Analysis

We conducted the statistical analysis from August 1, 2019, to January 11, 2020. We used descriptive statistics to compare differences between the groups for clinical and demographic characteristics. We compared between-group differences in symptom scores from baseline to 6 and 12 months using mixed-effect generalized linear regression models for repeated measures. We compared survival using Kaplan-Meier methods and risk of death using Cox proportional hazards regression models. We compared health care use (ED and hospitalizations) per patient using Poisson regression models with an offset term for length of follow-up and normalized ED visits and hospital admission rates per 1000 members per year. We compared health care costs using generalized linear regression models with a gamma link-log function to account for skewed data with an offset term for length of follow-up. All models were adjusted for age, sex, stage of disease, cancer diagnosis, and risk adjustment factor. All models included random effects to account for clustering of the individual within the clinic site.^23^ All *P* values were from 2-sided tests, and the results were deemed statistically significant at *P* = .05. We used Stata, version 15.1 (StataCorp)^24^ to conduct all analyses. Regression model results are presented as between-group differences with 95% CIs. We accounted for a 10-month administrative delay in claims.

## Results

**Figure 1.**
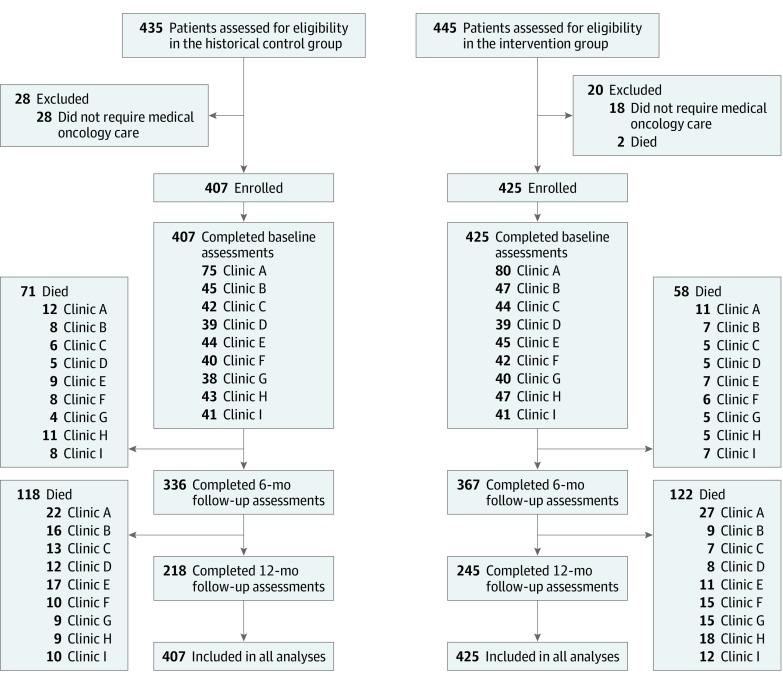
Assessment for Eligibility and Follow-up Flow diagram of patient flow through the study.

A total of 407 patients were identified in the control group and 425 patients participated in the intervention (Figure 1). There were no patients who dropped out of the study or who were lost to follow-up (Figure 1). There were no differences in age, race/ethnicity, cancer diagnosis, cancer stage, or risk adjustment factor between the groups across clinics; however, clinic A had a higher proportion of male patients in the control group compared with the intervention group (50 of 75 [66.7%] vs 42 of 80 [52.5%]; Table 1 and Table 2).

**Table 1.** Baseline Characteristics of Study Participants in Clinics A, B, C, D, and E^a^

Variables	Clinic A			Clinic B			Clinic C			Clinic D			Clinic E		
	CON	INT	*P* Value	CON	INT	*P* Value	CON	INT	*P* Value	CON	INT	*P* Value	CON	INT	*P* Value
Patients, No.	75	80		45	47		42	44		39	39		44	45	
Sex															
Male	50 (66.7)	42 (52.5)	.07	28 (62.2)	24 (51.1)	.28	30 (71.4)	24 (54.5)	.11	21 (53.8)	27 (69.2)	.16	28 (63.6)	25 (55.6)	.43
Female	25 (33.3)	38 (47.5)		17 (37.7)	23 (48.9)		12 (28.6)	20 (45.5)		18 (46.2)	12 (30.8)		16 (36.4)	20 (44.4)	
Age, mean (SD), y	78.6 (1.1)	79.6 (1.0)	.50	79.3 (1.4)	79.5 (1.2)	.96	80.0 (1.3)	78.1 (1.2)	.26	80.0 (1.2)	77.1 (1.5)	.11	80.7 (1.1)	78.9 (1.4)	.31
Race/ethnicity^b^															
Non-Hispanic white	36 (48.0)	38 (47.5)	.49	18 (40.0)	25 (53.2)	.50	22 (52.4)	25 (56.8)	.92	20 (51.3)	16 (41.0)	.71	15 (34.2)	18 (40.0)	.27
Hispanic	28 (37.3)	37 (46.3)		21 (46.7)	18 (38.3)		17 (40.4)	17 (38.6)		15 (38.5)	20 (51.3)		24 (54.5)	19 (42.2)	
Non-Hispanic black	6 (8.0)	3 (3.8)		3 (6.7)	3 (6.4)		1 (2.4)	1 (2.3)		1 (2.6)	1 (2.6)		2 (4.5)	1 (2.2)	
Asian Pacific Islander	3 (4.0)	1 (1.3)		1 (2.2)	1 (2.1)		2 (4.8)	1 (2.3)		2 (5.1)	2 (5.1)		3 (6.8)	3 (6.7)	
Native Hawaiian, Alaskan Native, or American Indian	2 (2.7)	1 (1.3)		2 (4.4)	0		0	0		1 (2.5)	0		0	4 (8.9)	
Cancer diagnosis															
Thoracic	6 (8.0)	8 (10.0)	.57	5 (11.1)	7 (14.9)	.99	4 (9.5)	4 (9.1)	.79	4 (10.3)	5 (12.8)	.26	5 (11.4)	1 (2.2)	.06
Gastrointestinal	19 (25.3)	19 (23.7)		12 (26.7)	10 (21.3)		9 (21.4)	7 (15.9)		11 (28.2)	10 (25.7)		10 (22.7)	15 (33.3)	
Head and neck	3 (4.0)	4 (5.0)		2 (4.4)	3 (6.4)		0	1 (2.3)		1 (2.6)	0		2 (4.6)	1 (2.2)	
Malignant hematologic	6 (8.0)	4 (5.0)		6 (13.3)	7 (14.9)		7 (16.7)	4 (9.1)		4 (10.3)	1 (2.6)		6 (13.6)	5 (11.1)	
Genitourinary	8 (10.7)	17 (21.3)		4 (8.9)	4 (8.5)		4 (9.5)	5 (11.4)		8 (20.5)	3 (7.7)		4 (9.1)	9 (20.0)	
Other^c^	9 (12.0)	9 (11.3)		7 (15.6)	6 (12.7)		4 (9.5)	8 (18.2)		2 (5.1)	7 (17.9)		3 (6.8)	8 (17.8)	
Breast	24 (32.0)	19 (23.7)		9 (20.0)	10 (21.3)		14 (33.4)	15 (34.1)		9 (23.0)	13 (33.3)		14 (31.8)	6 (13.3)	
Cancer stage at diagnosis															
I	13 (17.3)	14 (17.5)	.77	14 (31.1)	11 (23.4)	.29	13 (30.9)	13 (29.6)	.65	5 (12.8)	7 (17.9)	.89	9 (20.5)	9 (20.0)	.62
II	16 (21.3)	16 (20.0)		5 (11.1)	9 (19.1)		6 (14.3)	11 (25.0)		8 (20.5)	6 (15.4)		8 (18.2)	8 (8.9)	
III	12 (16.0)	18 (22.5)		4 (8.9)	9 (19.1)		7 (16.7)	6 (13.6)		11 (28.2)	11 (28.2)		8 (18.2)	10 (22.2)	
IV-VI	34 (45.4)	32 (40.0)		22 (48.9)	18 (38.3)		16 (38.1)	14 (31.8)		15 (38.5)	15 (38.5)		19 (43.2)	22 (48.9)	
RAF score, mean (SD)^d^	2.69 (1.86)	2.96 (1.86)	.37	2.89 (2.08)	2.54 (1.37)	.34	2.67 (1.70)	2.23 (1.65)	.22	2.69 (1.74)	2.42 (1.69)	.24	2.55 (1.99)	2.59 (1.75)	.92

Abbreviations: CON, control; INT, intervention; RAF, risk adjustment factor.

^a^ Data are number (percentages) of patients unless otherwise indicated.

^b^ Racial/ethnic group was self-reported by patient.

^c^ Skin, brain, bone, soft tissue, or head and neck.

^d^ The Center for Medicare & Medicaid Services’ Hierarchical Condition Category risk adjustment model assigns a risk score, also called the RAF score, to each eligible beneficiary. A beneficiary’s RAF is based on health conditions the beneficiary may have, as well as demographic factors such as Medicaid status (defined as having ≥1 month of Medicaid eligibility during the base year), sex, and elderly or disabled status.

**Table 2.** Baseline Characteristics of Study Participants in Clinics F, G, H, and I^a^

Variables	Clinic F			Clinic G			Clinic H			Clinic I		
	CON	INT	*P* Value	CON	INT	*P* Value	CON	INT	*P* Value	CON	INT	*P* Value
Patients, No.	40	42		38	40		43	47		41	41	
Sex												
Male	21 (52.5)	22 (52.4)	.99	17 (44.7)	25 (62.5)	.12	30 (69.8)	24 (51.1)	.07	28 (68.3)	21 (51.2)	.12
Female	19 (47.5)	20 (47.6)		21 (58.3)	15 (37.5)		13 (30.23)	23 (48.9)		13 (31.7)	20 (48.8)	
Age, mean (SD), y	79.4 (1.3)	80.0 (1.2)	.74	77.7 (1.2)	77.5 (1.4)	.93	77.9 (1.2)	76.4 (1.1)	.34	78.9 (1.2)	77.2 (1.1)	.30
Race/ethnicity^b^												
Non-Hispanic white	23 (57.5)	20 (47.6)	.08	22 (57.8)	21 (52.5)	.14	21 (48.8)	22 (46.8)	.41	22 (53.7)	23 (56.1)	.89
Hispanic	11 (27.5)	21 (50.0)		12 (31.6)	19 (47.5)		17 (39.5)	21 (44.7)		16 (39.0)	14 (34.2)	
Non-Hispanic black	1 (2.5)	1 (2.4)		0	0		1 (2.3)	0		1 (2.4)	1 (2.4)	
Asian Pacific Islander	3 (7.5)	0		2 (5.3)	0		2 (4.7)	0		2 (4.9)	2 (4.9)	
Native Hawaiian, Alaskan Native, or American Indian	2 (5.0)	0		2 (5.3)	0		2 (4.7)	4 (8.5)		0	1 (2.4)	
Cancer diagnosis												
Thoracic	3 (7.5)	7 (16.7)	.73	3 (7.8)	3 (7.5)	.99	3 (7.0)	3 (6.4)	.08	6 (14.6)	5 (12.2)	.14
Gastrointestinal	13 (32.5)	10 (23.8)		13 (34.2)	16 (40.0)		16 (37.2)	20 (42.6)		13 (31.7)	10 (24.4)	
Head and neck	1 (2.5)	2 (4.8)		0	0		0	3 (6.4)		1 (2.4)	3 (7.3)	
Malignant hematologic	3 (7.5)	4 (9.5)		5 (13.2)	4 (10.0)		4 (9.3)	8 (17.0)		4 (9.8)	5 (12.2)	
Genitourinary	5 (12.5)	4 (9.5)		7 (18.4)	6 (15.0)		1 (2.3)	3 (6.4)		1 (2.4)	6 (14.6)	
Other^c^	3 (7.5)	5 (11.9)		2 (5.3)	3 (7.5)		4 (9.3)	2 (4.3)		4 (9.8)	7 (17.1)	
Breast	12 (30.0)	10 (23.8)		8 (21.1)	8 (20.0)		15 (34.9)	8 (17.0)		12 (29.3)	5 (12.2)	
Cancer stage at diagnosis												
I	12 (30.0)	11 (26.2)	.90	5 (13.2)	10 (25.0)	.47	11 (25.6)	12 (25.5)	.99	9 (21.9)	5 (12.2)	.30
II	7 (17.5)	7 (16.7)		5 (13.2)	5 (12.5)		9 (20.9)	10 (21.3)		5 (12.2)	11 (26.8)	
III	6 (15.0)	5 (11.9)		7 (18.4)	9 (22.5)		6 (14.0)	7 (14.9)		8 (19.5)	6 (14.6)	
IV-VI	15 (37.5)	19 (45.2)		21 (55.2)	16 (40.0)		17 (39.5)	18 (38.3)		19 (46.4)	19 (46.4)	
RAF score, mean (SD)^d^	2.76 (1.66)	2.22 (1.82)	.16	2.56 (1.73)	2.84 (2.07)	.52	2.46 (1.22)	2.73 (2.12)	.77	2.83 (2.23)	2.93 (1.79)	.81

Abbreviations: CON, control; INT, intervention; RAF, risk adjustment factor.

^a^ Data are number (percentages) of patients unless otherwise indicated.

^b^ Racial/ethnic group was self-reported by patient.

^c^ Skin, brain, bone, soft tissue, or head and neck.

^d^ The Center for Medicare & Medicaid Services’ Hierarchical Condition Category risk adjustment model assigns a risk score, also called the RAF score, to each eligible beneficiary. A beneficiary’s RAF is based on health conditions the beneficiary may have, as well as demographic factors such as Medicaid status (defined as having ≥1 month of Medicaid eligibility during the base year), sex, and elderly or disabled status.

### Process Metrics

There were no deviations to the intervention processes during the study. The LHW contacted all patients 1 week prior to their first oncology visit. The mean (SD) number of LHW-patient contacts was 32.8 (11.2) (eAppendix 2 in the Supplement).

### Symptom Scores

**Figure 2.**
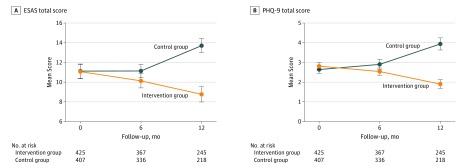
Mean Edmonton Symptom Assessment System (ESAS) Scores and Mean Patient Health Questionnaire-9 (PHQ-9) Scores A, Mean ESAS scores are measured on a numerical scale of 0 (absent) to 10 (worse). B, Mean PHQ-9 scores are measured on a numerical scale of 0 (not at all) to 3 (nearly every day) and scored from 0 to 27 where a score of 5 is a cutpoint for mild depression, a score of 10 is a cutpoint for moderate depression, a score of 15 is a cutpoint for moderately severe depression, and a score of 20 is a cutpoint for severe depression. *P* < .001 for difference in change in mean symptom scores between groups from baseline to 12-month follow-up.

Figure 2 depicts mean symptom scores by group at baseline and 6 and 12 months after diagnosis. The patients in the intervention group experienced reductions in symptoms over time (mean [SD] change in total ESAS score, −1.9 [14.2]; 95% CI, −3.77 to −0.19; *P* = .01), whereas the patients in the control group experienced worsening symptoms over time (mean [SD] change in total ESAS score, 2.32 [17.7]; 95% CI, 0.47-4.19; *P* = .02). The mean [SD] between-group differences were statistically significant (–4.75 [1.01]; 95% CI, –6.72 to –2.78; *P* = .02). With the exception of drowsiness and appetite (which did not change significantly among patients in either group), the patients in the intervention group experienced significant reductions in all symptoms compared with the patients in the control group over time (eFigure 1 in the Supplement). The patients in the intervention group also experienced significant reductions in the severity of depression (change in mean [SD] total PHQ-9 scores, −0.63 [3.99]; 95% CI, −1.23 to −0.03; *P* = .04); however, the patients in the control group experienced an increase in mean PHQ-9 scores over time (change in mean [SD] total PHQ-9 scores, 1.67 [5.49]; 95% CI, 0.95-2.37; *P* = .01) (Figure 2). These mean (SD) between-group differences were statistically significant (–2.07 [0.37]; 95% CI, –2.80 to –1.34; *P* = .001).

### Survival

At 12-month follow-up, there were no differences in median survival across both groups, with 180 of 425 patients (42.4%) having died among the intervention group and 189 of 407 patients (46.4%) having died among the control group (eFigure 2 in the Supplement). In Cox proportional hazards regression models, there were no differences in the risk of death after adjusting for age, stage of cancer, sex, cancer diagnosis, and risk adjustment factor across groups (hazard ratio for intervention vs control, 0.92; 95% CI, 0.75-1.13; *P* = .41).

### Health Care Use and Total Health Care Costs

Table 3 reports differences in health care use and costs between groups. The patients in the intervention group experienced greater use of palliative care (207 of 425 [48.7%] vs 101 of 407 [24.8%]; odds ratio, 2.83; 95% CI, 1.05-7.93; *P* = .04) and behavioral medicine (126 of 425 [29.6%] vs 46 of 407 [11.3%]; odds ratio 3.41; 95% CI, 2.76-4.21; *P* < .001) compared with the patients in the control group. The patients in the intervention group experienced fewer ED visits (mean [SD], 0.43 [0.76]; 95% CI, 0.36-0.50 vs 0.57 [1.00]; 95% CI, 0.48-0.67; *P* = .002) and hospitalizations (mean [SD], 0.54 [0.77]; 95% CI, 0.47-0.61 vs 0.72 [1.12]; 95% CI, 0.61-0.83; *P* = .04) per 1000 members per year compared with the patients in the control group. The patients in the intervention group also experienced lower median total costs ($17 869 [interquartile range, $6865-$32 540] vs $18 473 [interquartile range, $6415-$37 910]; *P* = .02) compared with the patients in the control group.

**Table 3.** Data on Health Care Use

Variable	Usual Care	Intervention	*P* Value
Total costs of care^a^			
Palliative care use, No. (%)	101 (24.8)	207 (48.7)	.04^b^
Behavioral medicine use, No. (%)	46 (11.3)	126 (29.6)	<.001^b^
No. of acute care visits per 1000 members/y, mean (SD)			
Emergency department use	0.57 (1.00)	0.43 (0.76)	.002^c^
Hospitalization use	0.72 (1.12)	0.54 (0.77)	.04^c^
Total health care costs, median (IQR), $	18 473 (6415-37 910)	17 869 (6865-32 540)	.02^d^
Last 30 d of life^e^			
No. of acute care visits, mean (SD)			
Emergency department use	0.30 (0.46)	0.10 (0.30)	.001^c^
Hospitalization use	0.43 (0.82)	0.27 (0.44)	.02^c^
Hospice care received, No. (%)	79 (41.2)	125 (69.4)	<.001^b^
Total health care costs, median (IQR), $	12 726 (5259-22 170)	3602 (1076-9436)	.002^d^
Acute care facility deaths, No. (%)	30 (15.9)	18 (10.0)	.14^b^

Abbreviation: IQR, interquartile range.

^a^ There were 425 patients in the intervention group and 407 patients in the control group. All data reported for within 12 months after enrollment. All models included random effects for the clinic site. All models were adjusted for age, sex, race/ethnicity, cancer diagnosis, cancer stage, and risk adjustment factor.

^b^ Estimated using logistic regression models.

^c^ Estimated using exact Poisson regression models with an offset term for length of follow-up.

^d^ Estimated using generalized linear model with a gamma link-log function to account for skewed data with an offset term for length of follow-up.

^e^ There were 189 patients in the intervention group and 180 patients in the control group who died. All data reported for within 12 months after enrollment. All models included random effects for clinic site. All models were adjusted for age, sex, race/ethnicity, cancer diagnosis, cancer stage, and risk adjustment factor.

### Health Care Use and Total Costs of End-of-Life Care

Among 189 patients in the control group and 180 patients in the intervention group who died within 12 months after diagnosis, there were significant differences in health care use and total costs at the end of life (Table 3). The patients in the intervention group experienced fewer ED visits (mean [SD], 0.10 [0.30]; 95% CI, 0.06-0.14 vs 0.30 [0.46]; 95% CI, 0.24-0.38; *P* = .001) and hospitalizations (0.27 [0.44]; 95% CI, 0.21-0.34 vs 0.43 [0.82]; 95% CI, 0.32-0.55; *P* = .02) compared with the patients in the control group. A greater proportion of patients in the intervention group than patients in the control group used hospice care (125 of 180 [69.4%] vs 79 of 189 [41.8%]; odds ratio, 3.16; 95% CI, 2.13-4.69; *P* < .001). The proportion of acute care facility deaths was not significantly different between intervention and control patients (18 of 180 [10.0%] vs 30 of 189 [15.9%]; odds ratio, 0.58; 95% CI, 0.28-1.20; *P* = .14). In the last 30 days of life, patients in the intervention group experienced lower median total costs compared with patients in the control group ($3602 [interquartile range, $1076-$9436] vs $12 726 [interquartile range, $5259-$22 170]; *P* = .002). There were also significant differences in the distribution of end-of-life total costs between the 2 groups (eFigure 3 in the Supplement).

## Discussion

This multisite LHW-led proactive symptom screening intervention for patients with newly diagnosed cancer was associated with lower symptom burden, acute care use, and total costs. The intervention was also associated with better quality of end-of-life care, less acute care use, and lower total end-of-life costs. Our findings of lower symptom burden associated with proactive symptom screening are similar to previous work^17^ and consistent with the literature.^1,25,26^ In this intervention, we expanded the LHW role to refer patients to palliative care and behavioral medicine in response to worsening symptoms and found improved symptoms, lower acute care use, and lower total costs within 1 year of diagnosis and at the end of life compared with a control group that received no standardized symptom screening interventions.

Undertreated symptoms are associated with prolonged hospitalizations and acute care readmissions.^10^ At the end of life, we found less health care cost variation among patients in the intervention group. Our findings may reflect associated lower acute care use and hospitalization length among patients in the intervention group due to lower symptom burden but could also reflect uniformity of services delivered, given that variations in end-of-life costs are often due to differing combinations of services provided in the acute care setting.^27^ More investigations are required to understand these findings.

Our study differs from technology-based approaches such as electronic portals or mobile phone–based symptom-reporting applications.^28,29,30^ These approaches can create barriers for patients, such as older adults, who may not prefer or be as comfortable with the use of technology-based health approaches.^31^ Furthermore, we chose to use a personnel-based approach based on patient and caregiver feedback.^13^ Unlike other personnel-led interventions that use health care professionals,^2,25,26^ we trained an LHW and expanded the intervention to all patients regardless of their cancer stage. Compared with studies using a lay navigation program,^32^ our intervention focused on symptom screening and referral and assessed their associations with health care use and cost and, perhaps more important, symptom burden over time. Although effective in advance care planning,^33^ LHW-led symptom screening and proactive referral to supportive care throughout the care continuum is unique. It is possible that our approach may have unmeasured benefits that technology-based approaches may lack. These unmeasured aspects, such as social support and counseling,^34^ in the LHW-patient relationship could be important in understanding the greater use of palliative care, behavioral medicine, and hospice care that we report. Future studies should evaluate these aspects.

Another unique aspect of our study was our collaboration with a payer that financially supported the implementation and community oncology practices that delivered the intervention. This stakeholder-engaged initiative^13,14,15^ allowed us to design benefits that could meet the needs of the patients, clinicians, and payers involved. One such example was the preauthorization for all LHW referrals to palliative care and behavioral medicine. By collaborating with the payer to bypass impediments to care, such as insurance authorization, patients in the intervention group could access timely and appropriate care for their reported symptoms. Another example is the $150 per member per month incentive from the payer to the oncology practice that enabled financial reimbursement for additional time incurred by the practice to conduct the intervention. Although early identification of symptoms was not associated with unscheduled clinic visits, given that the PA conducted all interventions via telephone, oncologists spent additional time daily reviewing LHW and supportive care services documentation and approving interventions conducted by the PA.

### Limitations

This study has some limitations. The patients in the intervention group were compared with a retrospective cohort of patients; thus, the causality of the intervention and our findings cannot be inferred. Although the demographic characteristics between the control and intervention groups were similar, there were differences, for example, by sex in 1 clinic. Although we are unaware of changes in practice patterns or secular trends during this time frame, it is possible that there could be differences in unmeasured variables independent of this intervention that could bias our findings. Second, we were unable to measure the PA interventions made in response to symptoms, which may be important in understanding our outcomes. Another limitation was the practice setting. We conducted the intervention among 9 community-based clinics in Los Angeles, California, that were associated with 1 cancer institute and limited the intervention to elderly CareMore Health Medicare Advantage beneficiaries. Thus, generalizability to other populations and organizations may be limited. Furthermore, 1 LHW and 1 PA conducted this intervention. It is possible that they may have been aware of the innovation being tested and optimized the intervention. The feasibility and effect of using multiple LHWs and supervising personnel needs to be further investigated. In addition, although we collected information on palliative care and behavioral medicine use, we did not measure LHW referrals, and therefore we were unable to assess the effect of the intervention on these intermediary variables. In addition, we did not adjust for multiple comparisons, which could have affected the *P* values we reported.

## Conclusions

This LHW-led symptom screening and referral intervention was associated with lower symptom burden, acute care use, and total costs, without survival differences between the intervention group and the control group. At the end of life, the intervention was associated with lower acute care use and total costs and greater hospice use. This intervention may be one approach to improve the value of cancer care delivery in community practices.

## Notes

Supplement. **eAppendix 1.** Intervention Workflow **eAppendix 2.** Lay Health Worker Program Process Metrics and Interactions **eFigure 1.** Mean Edmonton Symptom Assessment System Scores by Group at Baseline, 6- and 12-Months Follow-up **eFigure 2.** Survival Over 12 Months by Group **eFigure 3.** Distribution of Total Health Care Costs in the Last 30 days of Life by Group
