# Supplementary material for: Association of a Lay Health Worker Intervention With Symptom Burden, Survival, Health Care Use, and Total Costs Among Medicare Enrollees With Cancer
Source: JAMA Netw Open. 2020 Mar 16;3(3):e201023. doi: 10.1001/jamanetworkopen.2020.1023 (PMC7076340; doi:10.1001/jamanetworkopen.2020.1023)
Supplement: Supplement. — eAppendix 1. Intervention Workflow eAppendix 2. Lay Health Worker Program Process Metrics and Interactions eFigure 1. Mean Edmonton Symptom Assessment System Scores by Group at Baseline, 6- and 12-Months Follow-up eFigure 2. Survival Over 12 Months by Group eFigure 3. Distribution of Total Health Care Costs in the Last 30 days of Life by Group [file jamanetwopen-3-e201023-s001.pdf]

Supplementary Online Content

Patel MI, Ramirez D, Agajanian R, Agajanian H, Coker T. Association of a lay health worker intervention with symptom burden, survival, health care use, and total costs among Medicare enrollees with cancer. *JAMA Netw Open.* 2020;3(3):e201023. doi:10.1001/jamanetworkopen.2020.1023

eAppendix 1. Intervention Workflow
eAppendix 2. Lay Health Worker Program Process Metrics and Interactions
eFigure 1. Mean Edmonton Symptom Assessment System Scores by Group at Baseline, 6- and 12-Months Follow-up
eFigure 2. Survival Over 12 Months by Group
eFigure 3. Distribution of Total Health Care Costs in the Last 30 Days of Life by Group

This supplementary material has been provided by the authors to give readers additional information about their work.

© 2020 Patel MI et al. *JAMA Network Open.*

eAppendix 1. Intervention Workflow

Stanford Integrated Higher-Value Cancer Care
A Quality Improvement Program
Lay Health Worker Expanded Intervention Workflow

This workflow is only available to the patients in the intervention from 11/1/2016-10/31/2017. Patients in the control group received usual care which did not provide any of the below services.

Identifying Patients

- ❖ All patients with newly diagnosed cancer, except non-melanoma skin cancer, non-metastatic thyroid cancer, and prostate cancer not requiring Medical Oncology will be included in the lay health worker program

1) Patients will be categorized as either "Active" or "Inactive" based on the following criteria:
1) Active: All metastatic cancer patients and any non metastatic cancer patient on active chemotherapy
2) Inactive: Patients who are not on chemotherapy and are undergoing routine follow-up/surveillance
3) Three months after a non-metastatic patient has completed adjuvant chemotherapy, he/she will be transitioned from an "active" to "inactive" status unless trigger event occurs

Patient Engagement

- ➢ LAY HEALTH WORKER will contact all patients via telephone who meet the criteria for program prior to their first oncology appointment
- ➢ LAY HEALTH WORKER will schedule an initial oncology consultation appointment with the patient within 5 days of initial contact
- ➢ LAY HEALTH WORKER will discuss the program and goals
- ➢ LAY HEALTH WORKER will prepare the patient for their upcoming initial consultation with an oncologist
- ➢ LAY HEALTH WORKER will invite and encourage the patient to include anyone in the conversation such as family/caregiver and encourage the patient to include anyone who may potentially be attending future oncology appointments with the patient.
    - ➢ If necessary, the LAY HEALTH WORKER will offer to reschedule the call to accommodate patient’s request for others to be present during conversation.
- ▪ LAY HEALTH WORKER will provide contact information for patients to use in the event of any future triggers and provide a list of all the appropriate triggers to the patient and the care circle.
    - o Triggers Include:
    - ➢ Disease progression
    - ➢ Unexpected news regarding treatment effectiveness/side effects
    - ➢ Potential change of treatment
    - ➢ ED visit for cancer or cancer symptom control
    - ➢ If for any reason patient does not feel comfortable with current goals of care plan
    - ➢ Life situation changes in a manner that potentially impacts goals of care plan

© 2020 Patel MI et al. *JAMA Network Open.*

- Oncologist refers patient back to the LAY HEALTH WORKER
- Patient family members need education/clarification/support

Introduction of Program

- Explain the purpose of the conversation is to prepare the patient for their upcoming oncologist appointment.
  - The health worker is here to support the patient throughout this process of receiving education and making decisions
  - Provide educational materials regarding symptom assessment and referral services. Offer the patient the option to review educational material in their home (confirm home address).

- Identify symptom control resources, which the oncologist may later prescribe as appropriate (including palliative care resources).
  - *Note:* Here is a good place to address palliative care and begin the intake/ESAS. In addition, connect the patient with individual and group-based opportunities (support groups at the Cancer Institute) for emotional support involving interaction with trained professionals and other people undergoing similar health challenges

- Invite and encourage the patient to include anyone in the conversation such as family/caregiver and encourage the patient to include anyone who may potentially be attending future oncology appointments with the patient. Ask patient "are there any members of your family that you think will be part of your care?'
  - *Note*: If necessary, offer to reschedule the call to accommodate patient's request.
- Provide ESAS (**Please see referral to symptom call center algorithms and Palliative Medicine)
  - *Note:* The ESAS will be measured at routine intervals throughout the patient's journey in the symptom control call center as well based on the risk stratification categories listed above.
  - During the phone conversation use validated techniques using the PHQ9 to screen patients for need of psycho/social supportive services and refer to palliative care or psychosocial services.

Educational Materials

- All cancer patients will be educated on the general topics of chemotherapy, radiation, and surgical approaches to treating cancer.
- LAY HEALTH WORKER will discuss available handouts and reading materials with patients during the initial phone conversation

© 2020 Patel MI et al. *JAMA Network Open.*

- Front office staff will provide patients with handouts for their specific diagnosis, chemotherapy and symptom management (constipation, diarrhea, nausea, etc.) upon check out

Edmonton Symptom Assessment System (ESAS)

1) During the initial phone conversation the LAY HEALTH WORKER will screen patients for symptom management using Edmonton Symptom Assessment System (ESAS) tool (attached)
2) After the initial consultation, LAY HEALTH WORKER will contact and screen all active patients via telephone on a weekly basis and all inactive patients every month
3) LAY HEALTH WORKER will input data into Rabbit EHR via ESAS/PHQ-9 template
4) PA will review findings of ESAS results through EHR and if patient scores 4 or higher PA will notify the treating oncology practitioner for symptom management and the LAY HEALTH WORKER will refer patients to palliative care (see below)
5) Treating oncology practitioner must review/treat and place appropriate orders for any positive findings are noted on the ESAS
6) If needed PA will place necessary orders for referrals and notify the treating oncology practitioner via Rabbit e-mail
7) If patients have any issues and/or concerns, the LAY HEALTH WORKER will address them with the patients and if needed LAY HEALTH WORKER will notify PA as well as the treating oncology practitioner via Rabbit e-mail

Welcome Letter

- Front office staff will give patients a welcome letter outlining the program, services, and contact information during their initial visit
- LAY HEALTH WORKER will contact patients the day after their initial consultation to ensure they have received the welcome letter and document it in EHR
- In the event a patient did not receive a welcome letter, LAY HEALTH WORKER will mail one out to the patient's home

Care Plan

LAY HEALTH WORKER will ensure patient's procedures are being authorized and scheduled in a timely manner

- Referrals will be requested via the Utilization Management Provider Portal which have the following:
    1. Auto-approved Referrals:
        - Behavioral Health
        - Palliative Care
        - Questions contact the appropriate CCC the patient is scheduled

Patient Symptom Complaints/Triages

- When patients contact TOI staff with a symptom related complaint, TOI staff will create and assign a triage message to the appropriate mid-level provider per the most up to date TOI Mid-Level Triage schedule

© 2020 Patel MI et al. *JAMA Network Open.*

- The TOI employee creating the triage message must the PA-C" to the "Doctor" section of the triage message using the pre-set drop down menu in the center of the triage
- The treating oncology practitioner will then assess and treat the patient's symptoms per the symptom management algorithms provided to them
- PA will follow each triage message to ensure that the treating oncology practitioner is following all protocols
- Symptom Management Algorithms include:

- ❖ Diarrhea Treatment Algorithm
- ❖ Nausea/Vomiting Treatment Algorithm
- ❖ Constipation Treatment Algorithm
- ❖ Dyspnea Treatment Algorithm
- ❖ Pain Treatment Algorithm

<u>After Hour Calls</u>

- ➢ When a patient contacts TOI exchange after hours (between 5pm-8:30am Monday-Friday and all day Saturday/Sunday), the on-call treating oncology provider will be paged per the standing TOI protocol.
- ➢ All patients will be instructed to identify themselves as program patients to both the exchange service as well as to the treating oncology provider who takes the call.
- ➢ The treating oncology provider will assess and treat the patient's symptoms per the symptom management algorithms
- ➢ If the treating oncology provider feels that patient requires urgent evaluation which cannot wait until business hours, patient will be referred to the nearest emergency department.
- ➢ The treating oncology provider is required to notify the PA and LAY HEALTH WORKER about the call on the next business day. The message will include the reason for call, recommended treatment and outcome.
- ➢ PA will ensure that the treating oncology provider has followed symptom management algorithms
- ➢ PA will recommend more aggressive symptom management to the treating oncology practitioner if applicable and discuss follow-up plan with the LAY HEALTH WORKER

<u>All orders placed for referrals, procedures and imaging studies</u>

- ❖ Once an order has been placed for any procedure/referral, the front staff will process that order per standard TOI protocol.
- ❖ Front desk staff will notify the LAY HEALTH WORKER via Rabbit email of the order
- ❖ Front desk will notify the authorization department and scheduling department via Rabbit email of the order
- ❖ The authorization dept must obtain authorization from the payer in a timely manner for any of the services that are not already pre-authorized.
- ❖ The scheduler must schedule the patient the same day the authorization is received
- ➢ Medical records must obtain all appropriate reports prior to patient's next office visit
- ➢ If auth/scheduling/medical records have any issues they must notify the LAY HEALTH WORKER ASAP via Rabbit e-mail
- ➢ LAY HEALTH WORKER must review each patient's chart prior to each office visit to ensure patient has had all procedures done and all reports are in the chart

© 2020 Patel MI et al. *JAMA Network Open.*

Behavioral Health Referrals

- Patient should be considered for behavioral health referral if:
  - Patient scores 10 or higher on the PHQ-9
  - Patient has been diagnosed with Stage IV cancer (excluding lymphoma)
  - Patient is having difficulty coping with their diagnosis or treatment
- LAY HEALTH WORKER must notify the treating oncology practitioner regarding the referral for supportive services from TOI wellness center
- Front desk will process orders per standard protocol
- Front desk staff will notify the LAY HEALTH WORKER via Rabbit email of the referral status
- Front desk will notify authorization department and scheduling department via Rabbit email of the referral
- The authorization dept does not need authorization from the payer for behavioral health services
- The scheduler must schedule the patient the same day
- If scheduling departments encounter any issues with either process, they will notify the LAY HEALTH WORKER
- Representative from TOI wellness center will contact patient to discuss services available
- LAY HEALTH WORKER will follow up with patient and family to ensure that patient received the behavioral health consultation
- LAY HEALTH WORKER will ensure medical records department will obtain behavioral health consult report

Palliative Management Referrals

- Patient should be considered for Palliative Management referral if:
  - Patient's symptoms greater than 4 on ESAS
  - Patient symptoms change by 2 points
  - Patients report their symptoms are poorly controlled
  - Patient has numerous ED visits and hospitalizations due to poor symptom control
  - Patient is on high-dose opioid pain medications
  - Patient has severe symptoms not improving (7-10) despite non-pharmacologic and pharmacologic intervention
  - Patient has been diagnosed with Stage IV cancer (excluding lymphoma)
  - Patient needs treatment of the discomfort, symptoms and stress of serious illness
  - Patient has chosen supportive care treatment options but does not meet criteria for, or has declined, hospice care.
- LAY HEALTH WORKER will place referral for services from TOI wellness center.
- Front desk will process orders per standard protocol
- Front desk staff will notify the LAY HEALTH WORKER via Rabbit email of the referral status
- Front desk will notify authorization department and scheduling department via Rabbit email of the referral
- No authorization required from the payer as this is a pre-authorized service
- The scheduler must schedule the patient the same day the referral is received
- If scheduling departments encounter any issues with either process, they will notify the LAY HEALTH WORKER
- Representative from TOI wellness center will contact patient to discuss services available

© 2020 Patel MI et al. *JAMA Network Open.*

- LAY HEALTH WORKER will follow up with patient and family to ensure that patient received pain management consultation
- LAY HEALTH WORKER will ensure medical records department will obtain pain management consult report

Hospice Referrals

- Patient should be considered for hospice referral if:
  - Patient has a life expectancy of 6 months or less
  - Patient has advanced metastatic disease
  - Patient was noted to have progression of disease despite first or second line therapies where the burden of additional treatment outweighs the benefit.
  - Patient has cachexia or persistent, unintentional weight loss due progression of metastatic disease (>10% of body weight in the past 6 months)
  - Patient has a decline in performance status due to progression of metastatic disease (ECOG >2, Karnofsky score <50)
  - Patient has change in level of consciousness due to progression of metastatic disease
  - Patient has dyspnea with increased respiratory rate due to progression of metastatic disease
  - Patient has increasing ER visits and hospitalizations due to progression of metastatic disease
  - Patient wishes to discontinue aggressive cancer therapy and proceed with supportive care
  - Treating oncology practitioner will evaluate patient for hospice during office visit
  - PA will routinely review charts and notify treating oncology practitioner if PA determines patient is a candidate for hospice
  - Treating oncology practitioner will document discussion of hospice and if patient has agreed to or deferred hospice in EHR
- Once patient has been determined to be a hospice candidate and patient has agreed to hospice, referral consult should be placed.
- Front desk will process order per standard protocol
- Front desk staff will notify the LAY HEALTH WORKER via Rabbit email of the referral
- Front desk will notify authorization department and scheduling department via Rabbit email of the referral
- The authorization dept must obtain authorization from the payer in a timely manner
- The scheduler must schedule the patient the same day the authorization is received
- If authorization or scheduling departments encounter any issues with either process, they will notify the LAY HEALTH WORKER.
- LAY HEALTH WORKER will follow up with patient and family to ensure that hospice consultation was performed and will confirm if patient has agreed to or deferred hospice care.
    - If patient has agreed to hospice care, LAY HEALTH WORKER will notify the treating oncology practitioner as well as the PA-C, and Office Manager.
    - LAY HEALTH WORKER will also contact front desk and ask them to cancel all subsequent appts and mark the chart as a hospice patient.

Exit Criteria

- Patient Preference
  - If patient notifies staff of the Oncology Institute he/she does not wish to continue care, staff must place a triage the reason patient does not want to continue care and must notify LAY HEALTH WORKER and the treating oncology practitioner via Rabbit e-mail

© 2020 Patel MI et al. *JAMA Network Open.*

- If patient is leaving the practice due to being dissatisfied, LAY HEALTH WORKER will contact patient and discuss any issues/concerns
- Death
- Staff must follow standard office policy for documenting and marking the chart whenever a patient expires
- Staff must notify LAY HEALTH WORKER/PA-C as well as the treating oncology practitioner
- LAY HEALTH WORKER will ensure that chart has been appropriately documented in EHR

© 2020 Patel MI et al. *JAMA Network Open.*

eAppendix 2. Lay Health Worker Program Process Metrics and Interactions

All the patients assigned to the program (n=425) were enrolled and participated in the intervention. All phone calls with patients and the LHW were conducted between the hours of 8:30am and 5pm Monday through Friday. The LHW successfully contacted 100% of patients one week prior to their first oncology appointment. The LHW successfully reached 100% of all patients for subsequent symptoms assessments within 3 days of their assigned symptom assessment date. The mean (SD) number of contacts the lay health worker made with the patient for the duration of the 12-month study was 32.8 (11.2). Over the duration of the study, the average number of patients that the LHW followed actively was approximately 200.

Mean cost reductions for the intervention were $6,700 per patient in the intervention as compared to the control ($6,700 x 425) = $2,847,500. Intervention costs over the 12-month period were approximately $917,175 including personnel and provider incentives (LHW salary + benefits = $55,300; Physician Assistant salary + benefits = $96,875; $150 PMPM incentive x 12 months x 425 patients = $765,000). Thus the intervention reduced total expenditures by approximately 1.9 million US dollars.

© 2020 Patel MI et al. *JAMA Network Open.*

eFigure 1. Mean Edmonton Symptom Assessment System Scores by Group at Baseline, 6- and 12-Months Follow-up+

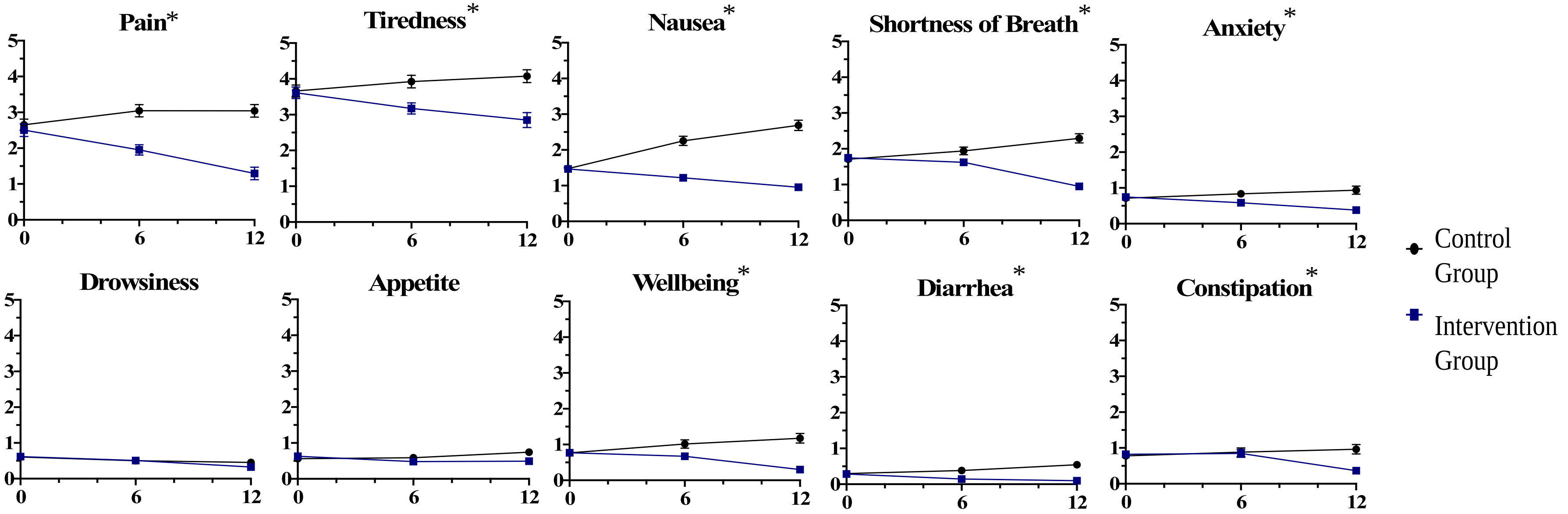

+Edmonton Symptom Assessment System is measured on a numerical scale of 0 (absent) to 10 (worse).
*p<0.001 for difference in change in adjusted mean symptom scores between groups from baseline to 12-months follow-up

© 2020 Patel MI et al. *JAMA Network Open.*

eFigure 2. Survival Over 12 Months by Group

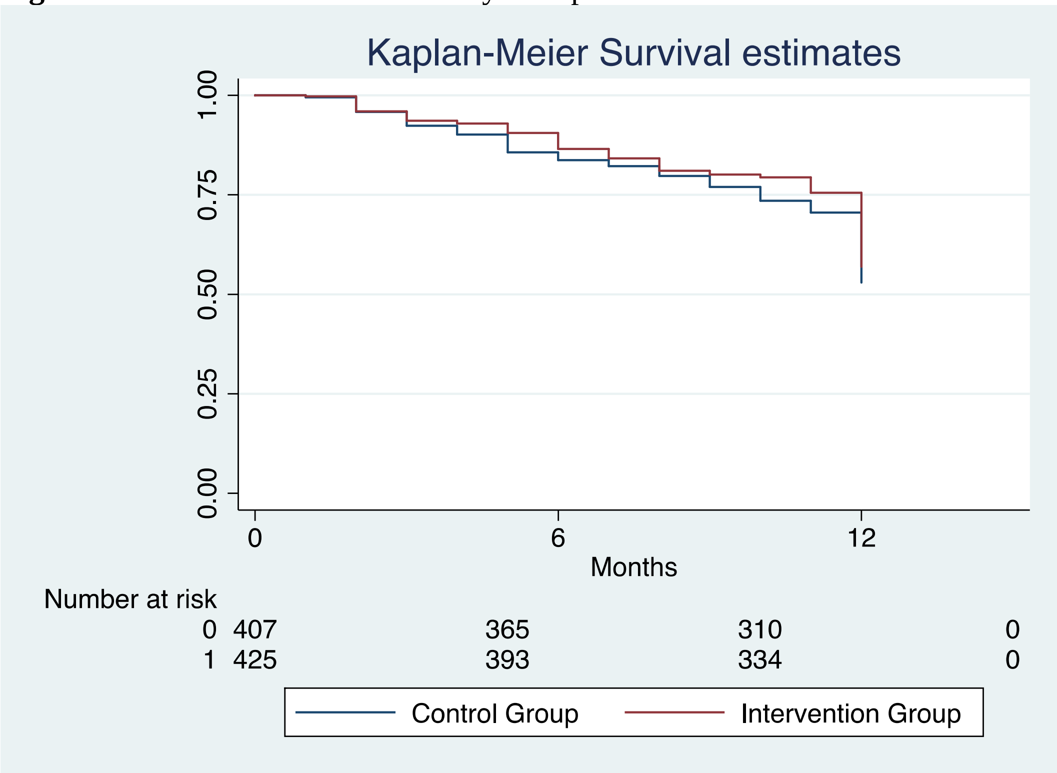

No differences in survival by group. Log-rank = 0.19

© 2020 Patel MI et al. *JAMA Network Open.*

eFigure 3. Distribution of Total Health Care Costs in the Last 30 Days of Life by Group

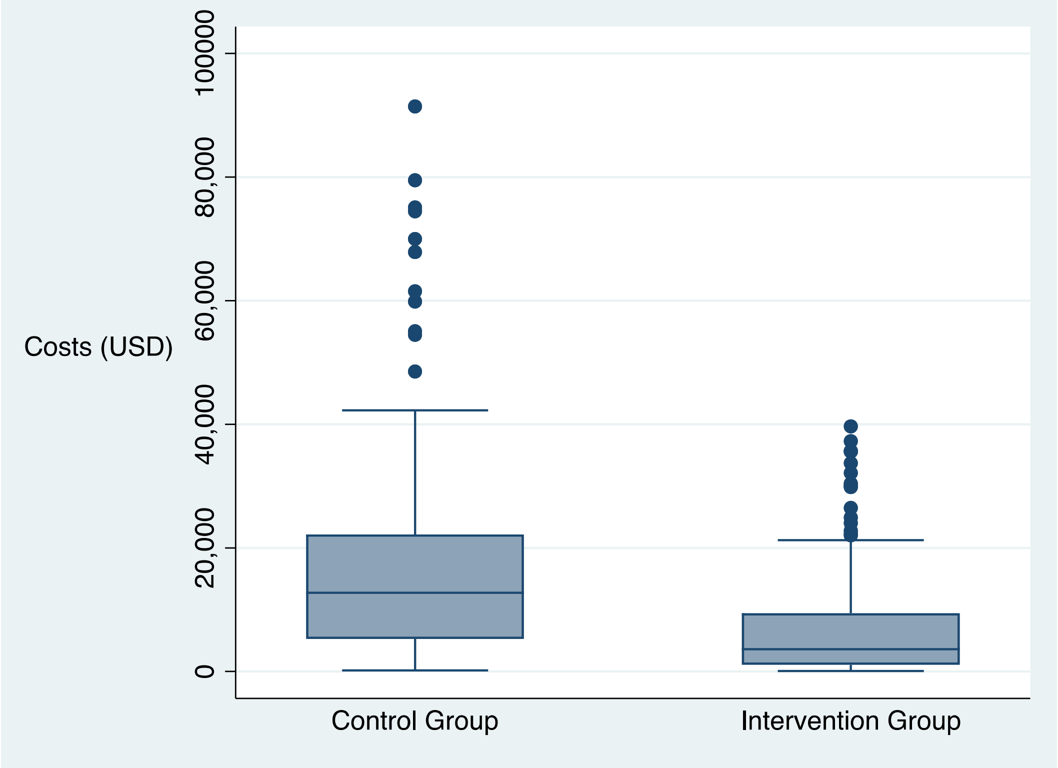

In this box plot, dots represent outliers. The maximum line represents the greatest total cost value excluding outliers. The upper quartile line represents 25% of data greater than this value. The middle line represents the median. The lower quartile line represents 25% of data less than this value. The minimum line represents the least value excluding outliers.

© 2020 Patel MI et al. *JAMA Network Open.*
